# Reviewer acknowledgement 2013

**DOI:** 10.1186/1752-2897-8-1

**Published:** 2014-03-03

**Authors:** Dirk Stengel, Annette Luckmann

**Affiliations:** 1 Unfallkrankenhaus Berlin, Berlin, Germany

## Abstract

**Contributing reviewers:**

The editors of the *Journal of Trauma Management and Outcomes* and BioMed Central would like to show our appreciation for the following reviewers for their time, hard work and support by reviewing manuscripts for the journal in 2013.

## 

Mazhar Abbas

India

Ibrahim Alhabdan

Saudi Arabia

Abdulbari Bener

Qatar

Ana Carlotti

Brazil

Charles Gerardo

United States of America

Morad Hameed

Canada

Manu Malbrain

Belgium

Gerrit Matthes

Germany

Jeffrey Indes

United States of America

Lynne Moore

Canada

Michael Pasquale

United States of America

Joao Rezende-Neto

Canada

Uli Schmucker

Germany

Vance Sohn

United States of America

Sandy Widder

Canada

Hiroshi Yatsushige

Japan

Niels Zorger

Germany

